# Systematic Analysis of Genes Related to Selenium Bioaccumulation in Microalgae: A Review

**DOI:** 10.3390/biology12050703

**Published:** 2023-05-12

**Authors:** Brenda S. Hoyos, Fabian Hernandez-Tenorio, Alejandra M. Miranda, Diego F. Villanueva-Mejía, Alex A. Sáez

**Affiliations:** 1 Biological Sciences and Bioprocesses Group, School of Applied Sciences and Engineering, Universidad EAFIT, Medellin 050022, Colombia; bhoyosg@eafit.edu.co (B.S.H.); ammirandap@eafit.edu.co (A.M.M.); dvillanu@eafit.edu.co (D.F.V.-M.); 2 Environmental Processes Research Group, School of Applied Sciences and Engineering, Universidad EAFIT, Medellin 050022, Colombia; fehernandt@eafit.edu.co

**Keywords:** microalgae, selenium, genes, bioaccumulation, systematic analysis

## Abstract

**Simple Summary:**

Selenium (Se) is an essential non-metallic nutrient in animals and humans. Its deficiency causes heart disease, infertility, neuronal/neuromuscular diseases, and increased susceptibility to cancer, infections, and heavy metal toxicity. Therefore, a sustainable strategy for bio-based products enriched with Se is microalgae. These are characterized by the ability to bioaccumulate inorganic Se and metabolize it into organic Se for product formulations of industrial interest. This review article attempts to elucidate the bioaccumulation of Selenium (Se) in microalgae from the analysis of genes or groups of genes that trigger biological responses associated with Se-metabolization in microalgae.

**Abstract:**

Se is one of the essential nutrients for human health and animal growth; it participates in various physiological functions, such as antioxidant and immune response and metabolism. Se deficiency is related in the animal industry to poor production performance and the appearance of health problems in humans. Therefore, interest has arisen in producing fortified foods, nutritional supplements, and animal feed products enriched with Se. A sustainable strategy for bio-based products enriched with Se is microalgae. These are characterized by the ability to bioaccumulate inorganic Se and metabolize it into organic Se for product formulations of industrial interest. Although there are some reports on Se bioaccumulation, further exploration is needed to understand the effects of Se bioaccumulation in microalgae. Therefore, this article presents a systematic review of the genes or groups of genes that trigger biological responses associated with the metabolization of Se in microalgae. A total of 54,541 genes related to Se metabolization distributed in 160 different classes were found. Similarly, trends were identified through bibliometric networks on strains of greatest interest, bioproducts, and scientific production.

## 1. Introduction

Se deficiency has been related to poor production performance in the animal industry (i.e., decreased weight gain, less milk and wool, fertility problems, size reduction, and low-quality semen) and health problems (i.e., degenerative lesions in the myocardium that increase pup deaths). Likewise, in humans, it has been associated with different forms of cancer, thyroid gland malfunction, and fertility problems, among others [1].

It has been found that in adequate concentrations, Se helps to positively support functions in different systems, such as the endocrine, immune, and cardiovascular systems [2,3]. In animal tissues, Se is found mainly in the form of selenocysteine and as selenomethionine in edible plants [4]. Once Se is available in the human body, it plays a role in around 30 selenium-dependent enzymes. These selenoenzymes are specifically involved in important biological processes such as metabolic pathways and the proper functioning of the immune system, thyroid hormone, and the triggering of antioxidant activities (i.e., glutathione peroxidase, which is associated with physiological and anticancer functions) [5]. For this reason, Se is an essential micronutrient. It is usually found in aquatic and terrestrial environments, and inorganic forms such as selenite (SeO_3_^2−^) and selenate (SeO_4_^2−^) in natural ranges of 0.01–0.5 mg Se/L [6], except in some toxic places contaminated by anthropogenic activities.

Despite its availability in the environment, it is challenging to meet Se requirements naturally through only the diet, especially in the animal industry [1]. Additionally, many factors affect the availability of minerals in forages or plants and bioaccumulation in aquatic environments [7]. Among them are the soil type, the presence of antagonistic and polluting elements, fertilization, climate and season, and the Se accumulation capacity of the species serving as a matrix. In addition, inorganic Se—as it is generally found in nature—is not an effective supplement. Several studies have shown that it is not easily absorbed and can be more toxic than other forms of organic Se [8,9]. Consequently, strategies are required to cover the daily Se requirement, which is 40 μg of Se for adults; 200 μg/day is considered as therapeutic and higher than 800 μg/day as toxic doses [10].

On the other hand, Se deficiency can put human population groups at risk, such as people with thyroid-related diseases, cancer, weakened immune functions, and pregnancy, among others [11,12]. Additionally, Se deficiency can have economic impacts on the animal industry, reducing the life quality of animals. Therefore, there is interest in producing Se-enriched fortified foods, nutritional supplements, and animal feed products.

Microalgae are among the strategies to contribute to developing edible products enriched with Se. Microalgae are a balanced source of proteins, lipids, vitamins, and trace elements already commercialized industrially as food supplements [13]. Additionally, they can capture and accumulate inorganic Se from the environment and metabolize a portion into organic Se, an eco-friendly and bio-based Se source. For example, it has been reported that the selenized biomass of *Chlorella vulgaris* has ~49% higher bioaccessibility, compared to that of yeast ~21%, and selenized supplements ~32%. However, further exploration is still needed to understand the effects of Se bioaccumulation on microalgal biomass [14]. Similarly, few investigations compare the effects of Se in the use of axenic cultures and co-cultures in order to improve Se bioaccumulation. Therefore, this article presents a systematic review of the genes or groups of genes that trigger biological responses associated with Se-metabolization in microalgae. Likewise, a bibliometric analysis of selenium bioaccumulation in microalgae was carried out, which focused on indicating trends in strains of greatest interest, bioproducts, and scientific production, among others. The analysis presented here represents an essential contribution from the systematic approach that could be useful for developing research on Se bioaccumulation in microalgae and could provide some suggestions for related research programs.

## 2. Methods

Systematic reviews were performed using scientometric tools that integrate statistical tools with information visualization techniques to explore structures and trends of subjects or research domains [15]. Scientometric analysis of selenium in microalgae may elucidate progress, development trends, and hotspots, as well as perspectives on Se bioaccumulation and genes related to Se metabolization in microalgae.

### 2.1. Bibliometric Analysis of Selenium Bioaccumulation in Microalgae

A systematic search was carried out in the Scopus scientific database under the search criteria established by Equation (1). The information collected was refined to avoid repeating terms with abbreviations and hyphens [16]. The bibliometric parameters, total number of citations, average citations per article, and categorization of the publications with the highest citations were calculated with the Bibiometrix software (University of Naples Federico II, Naples, Italy) from R Commander (×64. 4.1.0). The types of software used were VOSviewer version 1.6.17 (Leiden University, Leiden, The Netherlands) and CorText Manager (INRAE, Noisy-le-Grand, France) to develop bibliometric networks, such as Sankey diagram and Co-occurrence and maps of Co-authorship [17].

TITLE-ABS-KEY (“microalgae” AND (“selenium” OR “selenite” OR “selenate” OR “Se”)) AND (LIMIT-TO (DOCTYPE, “ar”) OR LIMIT-TO (DOCTYPE, “re”))(1)

### 2.2. Systematic Analysis of Genes Related to Selenium Metabolism

A systematic search for all genes associated with bioaccumulation, toxicity, or some other activity of selenium in cells was performed. The Gene platform of the National Center for Biotechnology Information (NCBI) of the United States was used. The search term was selenium * [Text Word]. Results for all three domains of life (archaea, bacteria, and eukaryotes) were included. The found genes were curated manually, and additional information was added, such as the class of organism to which each gene belonged. Once the data was curated, subsequent Data Science analyses were performed using R Studio 3.0.0 software and VOSViewer 1.6.17 (Leiden University, Leiden, The Netherlands).

## 3. Results and Discussion

### 3.1. Bibliometric Analysis of Selenium Bioaccumulation in Microalgae

#### 3.1.1. Scientific Production

**Figure 1:**
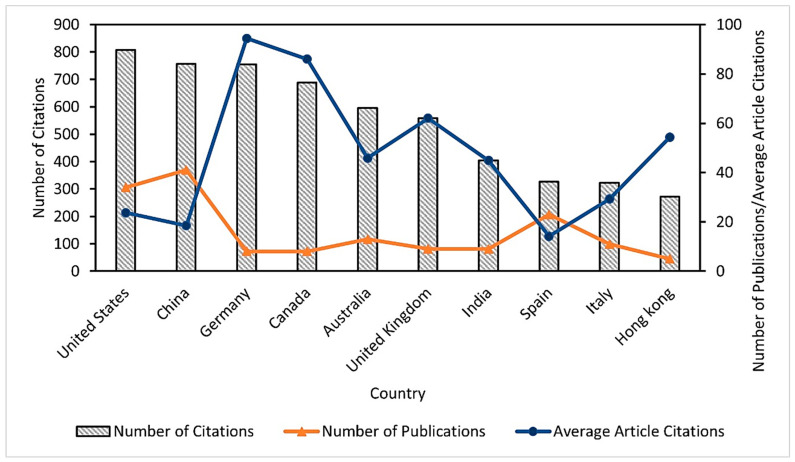
Leading countries in publications for the 1992–2022 period on selenium bioaccumulation in microalgae.

The bibliometric analysis allowed us to identify the relevance of the published research on selenium bioaccumulation in microalgae; consequently, the results showed the categorization of the leading countries in the publication of articles on the subject (Figure 1), finding that the United States of America (USA) presents the highest number of citations (807), followed by China (757). In addition, it was identified that Germany showed the highest average value of citations per article (94.37) and the lowest number of documents (8) compared to the USA (34) and China (41). Therefore, the impact of these publications in the study area and their possible use as important references for other research is evident. It should be noted that the information extracted from Scopus was categorized according to the affiliation address of the corresponding author.

#### 3.1.2. Sankey Diagram

**Figure 2:**
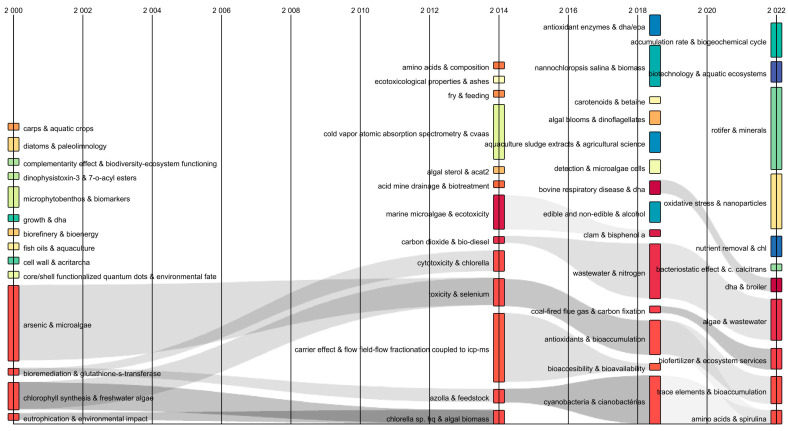
Sankey diagram of author’s keywords in publications on selenium bioaccumulation in microalgae.

The temporal flow of the keywords was analyzed using a Sankey diagram [18]. This identified transformations in keyword combinations over time. The diagram showed the interrelated keywords by flows of a gray color. The thickness represented the co-occurrences of the two keywords (Figure 2). Between 2000 to 2014, keyword combinations were identified: “arsenic and microalgae” and “bioremediation and glutathione-s-transferase” that converge with “toxicity and selenium”, while “chlorophyll synthesis and freshwater algae” and “eutrophication and environmental impact” converged with “*Chlorella* sp. and algal biomass”. This indicated that in 14 years, the study of microalgae transcended towards the “possible use” of selenium to “obtain enriched valuable biomass” to develop possible food supplements. Likewise, it was observed that in 2014–2018 the combination of “toxicity and selenium” converged with “antioxidants and bioaccumulation”. This was divided into the “trace elements and bioaccumulation” and “amino acids and spirulina” streams (2018–2022). Consequently, the Sankey diagram showed from the divergence and convergence of currents and the transformation of keywords showed the dynamic evolution of the research field over time [17].

#### 3.1.3. Co-Occurrences Analysis

**Figure 3:**
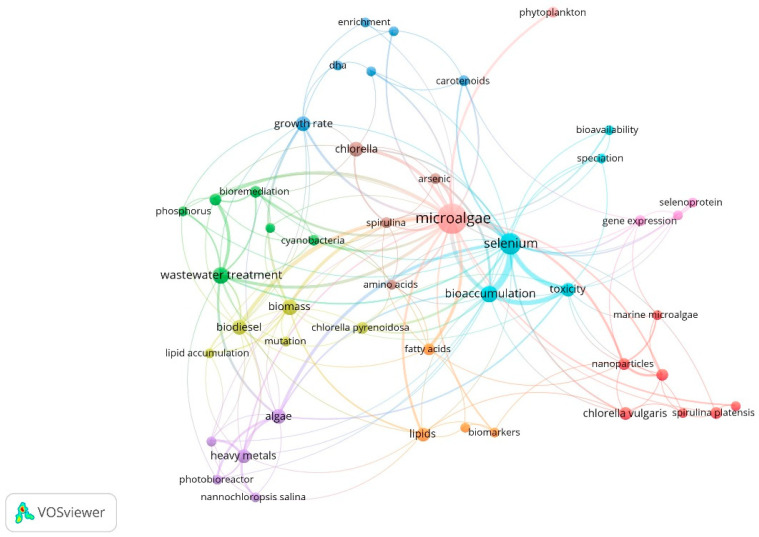
Bibliometric map of author keywords in publications on Se bioaccumulation in microalgae. Six theme groups: yellow, blue, green, purple, red, orange, violet, and sky blue.

Figure 3 shows the bibliometric map of author keywords obtained through the co-occurrence analysis. This shows eight groups of keywords are interrelated. The grouped themes are associated with strains of interest used due to their ability to metabolize Se (*Nannochloropsis salina*, *Chlorella pyrenoidosa*, *Chlorella vulgaris*, *Spirulina platensis*). Likewise, selenium-dependent proteins were identified, mainly those containing selenomethionine and selenocysteine residues. Additionally, terms related to biomolecules synthesized by microalgae, such as lipids, fatty acids, carotenoids, and amino acids, show the approach toward using microalgae biomass as alternatives for the energy and food sector. In addition, the term “genetic expression” was identified, which shows interest in understanding Se metabolization and the genes involved in the responses by microalgae under different Se concentrations. On the other hand, it was possible to establish the main reported keywords based on their co-occurrence: microalgae (71), selenium (30), bioaccumulation (14), wastewater treatment (14), and biomass (12) were the words used with greater proximity in published documents according to data from Scopus reports.

### 3.2. Systematic Analysis of Genes Related to Selenium Metabolism

Consistent with the effects of selenium from a macro-perspective, as shown above, a systematic gene search was performed. This was to analyze the potential genes that could be involved in the responses of microalgae when exposed to considerable concentrations of Se. The most significant number of species with genes related to Se metabolization was included in the systematic search. The reason is that in previous searches that were specific to *C*. *sorokiniana* and *S*. *obliquus*, it was determined that the information was scarce or almost non-existent. Therefore, it would be useful to know what was happening in other strains or species to detect potential similarities or differences with the group of interest that are microalgae. This systematic search was carried out on the NCBI platform, and a total of 54,541 genes were found, for which it has been reported that they are associated with Se metabolization.

**Figure 4:**
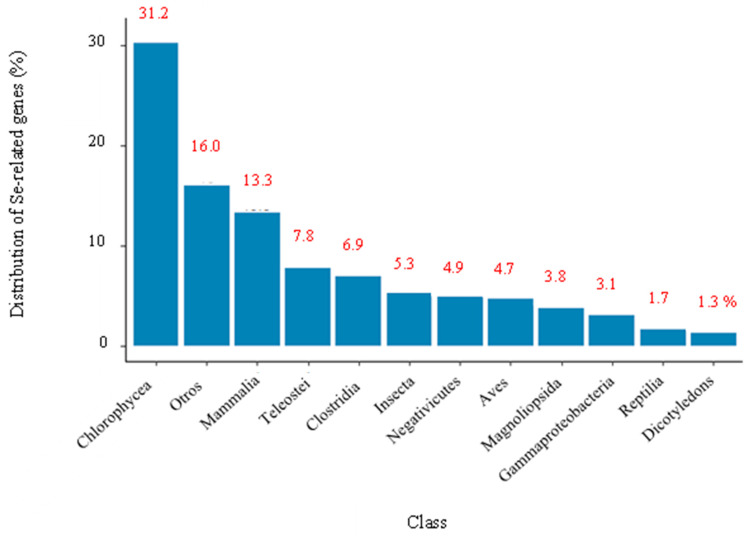
Distribution (%) of the 54,541 genes found through the systematic review—related to Se metabolization according to their class.

Manual curation and categorization were performed, and found that these genes belong to 160 different classes of organisms (Figure 4). The graph showed that Chlorophyceae, belonging to the green algae, was the class with the highest number of genes associated with Se, at 31.2%. While the class known as Dicotyledons, which are part of the flowering plants, was the one that presented the least amount of genes associated with Se (1.3%). This could indicate that green algae have the genetic potential to be used as Se biological tools. It should be noted that algae can be classified into two main groups; the first are the microalgae, which include blue–green algae, dinoflagellates, bacillariophytes (diatoms), etc., and the second are the macroalgae (seaweeds), which include green, brown, and red algae. In addition, they can be differentiated by size in unicellular formfrom 3–10 μm (microns) to giant algae up to 70 m long and growing up to 50 cm per day [19].

**Figure 5:**
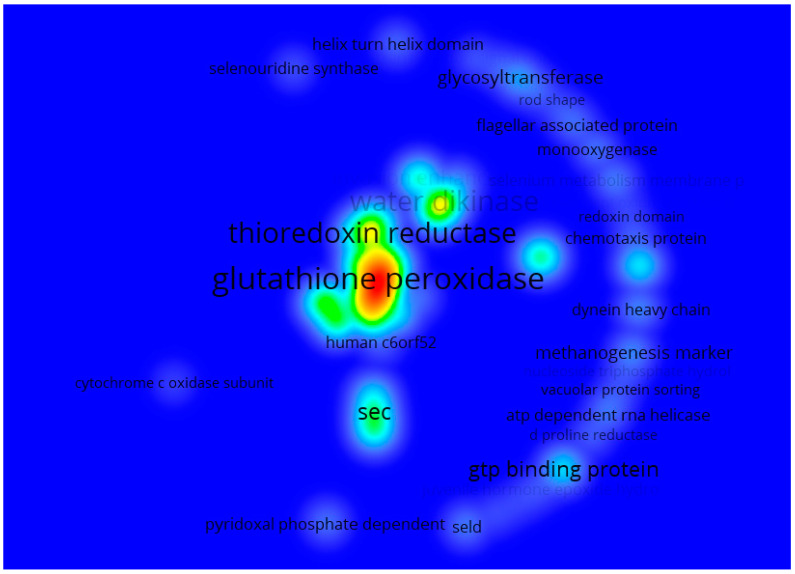
Heat map showing the terms primarily found in describing the 54,541 genes related to Se for 160 different classes of organisms. The red tones represent the most recurring terms, followed by yellow and green, while the less-recurring ones are denoted by cyan and finally blue. The font size is also proportional to how often the term can be found.

Likewise, Figure 5 shows that the most repeated terms were “glutathione peroxidase” (glutathione peroxidase), glutathione, “thioredoxin reductase” (thioredoxin reductase), “water dikinase” (water dikinase) and “sec”. All of which were an analysis of keyword co-occurrence of the annotations with the description of all 54,541 genes. The review found the relationship between these terms and selenium and glutathione peroxide (a key enzyme in cellular antioxidative defense systems and useful for detoxifying peroxides and hydroperoxides). Additionally, it is a selenium-dependent enzyme since it has selenocysteine in the enzyme’s catalytic site. Therefore, the availability of selenium regulates its enzymatic activity [20]. On the other hand, thioredoxin reductase is also a seleno-dependent protein, key for the metabolization of Se since it reduces the different chemical species of Se until it is made available for the synthesis of selenoproteins. Some studies have concluded that this type of enzyme is responsible for cells’ resistance to the cytotoxicity caused by Se [21]. In addition, aqua dikinase, an enzyme from the transferase family, can synthesize selenophosphate from selenide and ATP [22]. In the same way, the genes involved in synthesizing selenocysteine (sec) have a fundamental role concerning the metabolization of Se since they are required for selenoproteins’ biosynthesis, such as glutathione peroxide and thioredoxin reductase, previously mentioned. These are the ones that insert selenocysteine into proteins, which in turn will also play a crucial role in the metabolization of Se [23]. Therefore, it is possible to affirm that the genes found are probably more frequently repeated, related to the biosynthesis of enzymes involved in redox processes related to oxidative stress. This could indicate that in nature, a considerable number of species with biological mechanisms allow the metabolization or disposal of Se.

**Figure 6:**
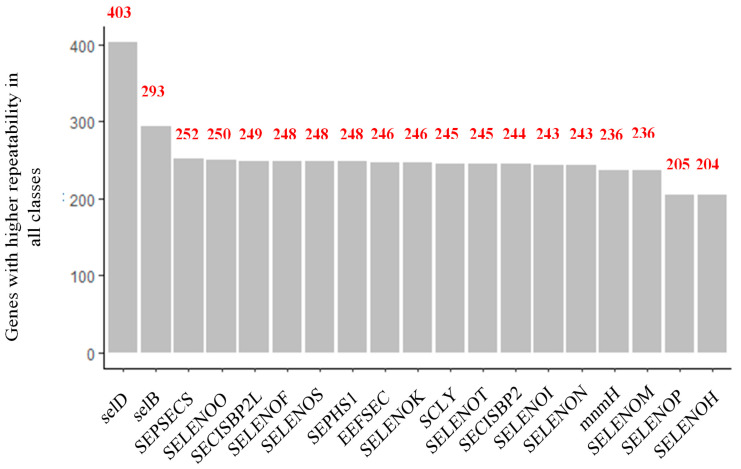
Genes repeated most frequently in all classes, with the 54,541 results obtained in the systematic review.

On the other hand, it was observed that, just as patterns were found in the description of all the genes found, some were also considerably repeated across all the species of the different classes (Figure 6). The gene with the highest co-occurrence was SelD, found 403 times, followed by SelB, SEPSECS, SELENOO, and SECISCBP2L, the latter showing co-occurrences of 293, 252, 250, and 249, respectively. From the literature, it was found that these most frequently found genes fulfill biological functions that could be described as basic and convenient, as summarized in Table 1. For example, SelD, SelB, and SEPSECS are elongation factors related to the synthesis and specific insertion of selenocysteine into proteins.

In the same way, it was found that six of these genes (SELENOF, SELENOK, SELENOM, SELENON, SELENOS, and SELENOT) have the particularity of coding for selenoproteins located in the endoplasmic reticulum (ER). It should be noted that the ER is responsible for protein synthesis, folding, modification, transport, and oxidative stress (in this case caused by excess Se) that occurs when its protein folding capacity becomes saturated and inefficient [24].

Other genes, such as SELENOO, SELENOI, SELENEP, and SELENOH, despite having a similar root to the previous ones, are located in other parts of the cells. In any case, they also have biological functions related to Se, such as biosynthesis of chemical species that contain Sec, decomposition reactions of Se to obtain elemental Se, and protection against cell damage, among others. These functions are similar to those fulfilled by SECISBP2L, SEPHS1, EEFSEC, SCLY, SECISBP2, and mnmH.

**Table 1:** Description of the biological function of the genes most repeated across all the different classes of organisms in the 54,541 results found in the systematic review.

Gene	Biological Function	Reference
SelD	This selenophosphate synthetase is highly specific for selenide, capable of discriminating sulfur. It has even been associated with regulating cell proliferation, growth, and differentiation.	[25,26]
SelB	It is a specialized translation elongation factor. Furthermore, it is an enzyme required for synthesizing and inserting selenocysteine into proteins.	[27]
SEPSECS	It provides instructions for making an O-phosphoseryl-tRNA (Sec) enzyme, which forms tRNAs critical to selenocysteine production.	[22]
SELENOO	It codes for a selenoprotein located in mitochondria and is known to be the largest selenocysteine-containing mammalian selenoprotein. However, the exact function of this is unknown, but it is believed to have redox activity.	[28]
SECISBP2L	It interacts with all known human SECIS (Sec insertion sequence) in vitro RNAs. Furthermore, selenoprotein mRNAs are co-immunoprecipitated with endogenous SBP2L, suggesting a role in regulating selenoprotein expression, but does not promote Sec incorporation into in vitro selenoproteins.	[29]
SELENOF	Due to S or Se, it could be involved in redox reactions associated with oxidative stress of the endoplasmic reticulum (ER) and carcinogenesis. In addition, it contributes to the quality control of protein folding in the ER. However, its biological function is still unclear.	[30]
SELENOS	It regulates inflammation, oxidative stress, and endoplasmic reticulum (ER) stress. It is also involved in the degradation process of misfolded proteins in the ER and could function as a modulator of Sec insertion.	[31]
SEPHS1	It encodes an enzyme that synthesizes selenophosphate from selenide and ATP. Selenophosphate is the selenium donor used to synthesize selenocysteine.	[32]
EEFSEC	It is a necessary translation factor for the incorporation of selenocysteine into proteins.	[28]
SELENOK	It is a transmembrane protein that participates in the ER-associated degradation of misfolded glycosylated proteins. It also has a role in protecting cells from stress-induced apoptosis in the ER. Furthermore, knockout studies in mice show the importance of this gene in promoting Ca^2+^ flux in immune cells and generating an effective immune response.	[33,34]
SCLY	Catalyzes the breakdown of L-selenocysteine into L-alanine and elemental selenium.	[35]
SELENOT	Studies in mice indicate a crucial role for this gene in the protection of dopaminergic neurons against oxidative stress in Parkinson’s disease and the control of glucose homeostasis in pancreatic beta cells.	[36]
SECISBP2	An essential component of the machinery involved in the co-translational insertion of selenocysteine (Sec) into selenoproteins. Mutations in this gene have been associated with reduced iodothyronine deiodinase type II enzyme activity (a selenoprotein) and abnormal thyroid hormone metabolism.	[37]
SELENOI	It is a multi-passage transmembrane protein. It catalyzes the transfer of phosphoethanolamine from CDP-ethanolamine to diacylglycerol to produce phosphatidylethanolamine, which is involved in forming and maintaining vesicular membranes, regulation of lipid metabolism, and protein folding.	[28]
SELENON	It plays an essential role in cell protection against oxidative stress and regulating calcium homeostasis in the ER related to redox activity.	[24]
mnmH	It codes for a selenophosphate-dependent tRNA 2-selenouridine synthase, essential for modifying some tRNAs to replace a sulfur atom with selenium. This enzyme works with SelD, the selenium donor protein, which also acts on the incorporation of selenocysteine.	[29]
SELENOM	Located in the perinuclear region, it is highly expressed in the brain and may be involved in neurodegenerative disorders. Studies have shown that transgenic mice with targeted deletion of this gene exhibit weight gain. That suggests a role for this gene in regulating body weight and energy metabolism.	[38,39]
SELENOP	It codes for a selenoprotein predominantly expressed in the liver and secreted into the plasma. This selenoprotein is unique because it contains multiple selenocysteine (Sec) residues per polypeptide (ten in humans) and accounts for most of the selenium in plasma. Mice lacking this gene exhibit neurological dysfunction.	[29]
SELENOH	Codes for a nucleolar selenoprotein. It functions as an oxidoreductase and has been shown to protect neurons against UVB-induced damage by inhibiting apoptotic cell death pathways. Promotes mitochondrial biogenesis and function and suppresses cellular senescence through genome maintenance and redox regulation.	[40]

**Figure 7:**
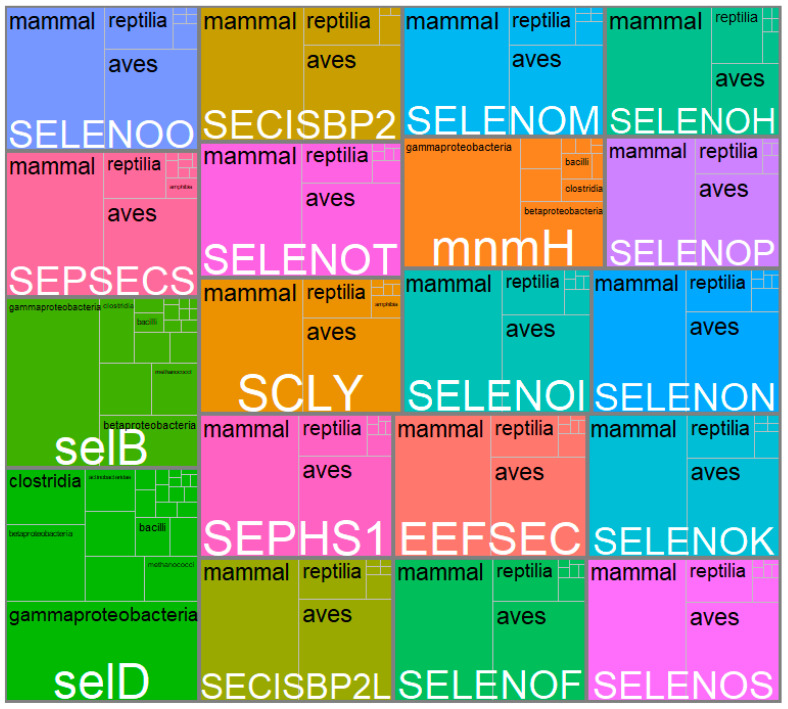
Treemap of the most frequently repeated genes and the main classes in which they were reported. Each large box belongs to a gene whose name is in white type. The sub-boxes within each box correspond to the number of classes and the black letter of these names. The sizes of the boxes, sub-boxes, and black letters are proportional to the number of times the gene was found in total and the number of times it was found in each class.

**Figure 8:**
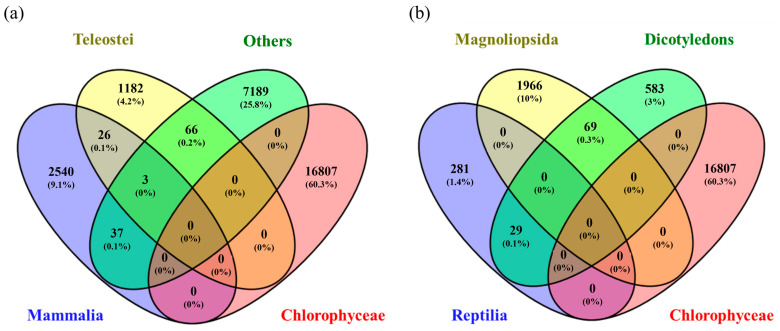
Venn diagram comparing the genes found for Chlorophyceae with (**a**) Teleostei, Others, and Mammalia and (**b**) Magnoliopsida, Dicotyledons, and Reptilia.

As shown in Figure 7, by reviewing in depth the classes to which these most frequently repeated genes belong, it was found that SelD, SelB, and mnmH were found mainly in different types of bacteria such as Gammaproteobacteria, Betaproteobacteria, Clostridia, and Bacilli. In contrast, the rest (SEPSECS, SELENOO and SECISCBP2L, SELENOF, SELENOS, SEPHS1, EEFSEC, SELENOK, SCLY, SELENOT, SECISBP2, SELENOI, SELENON, mnmH, SELENOM, SELENEP, and SELENOH) were found mainly in the classes Mammalia, Reptilia, and Birds. In other words, although 31.2% of all the genes found in the systematic review belonged to the Chlorophyceae class, others with a lower share of genes, such as Mammalia (13.3%), Clostridia (6.9%), Aves (4.7%), Gammaproteobacteria (3.1%) and Reptilia (1.7%) were the classes with highest genes shared, repeated most frequently. This could lead us to think that for Chlorophyceae, the largest number of genes related to Se metabolization was found. However, they differ throughout the species, hence the low gene repeatability. When comparing Chlorophyceae genes with each of the classes using a Venn diagram, it was found that they only shared 0.2% of genes with Teleostei, <0.01% with Mammalia, and 0.3% with Magnoliopsida, with the rest of the classes there were no genes in common (Figure 8).

Since no information was found in the literature, different hypotheses are suggested to explain these findings. This could be because this class’s genomes naturally have genetic variability and present various genes between species. Additionally, the research gaps have caused all the genomes of all the species that make up the Chlorophyceae class to not be fully known, which could cause genes of this class that are repeated throughout the others to be unknown. Even the gene naming system could cause similar genes to get confused because they have different names in other species. However, for the latter, a BLAST nucleotide alignment was made with the most frequently repeated genes, and no similar sequences were found in microalgae species.

**Figure 9:**
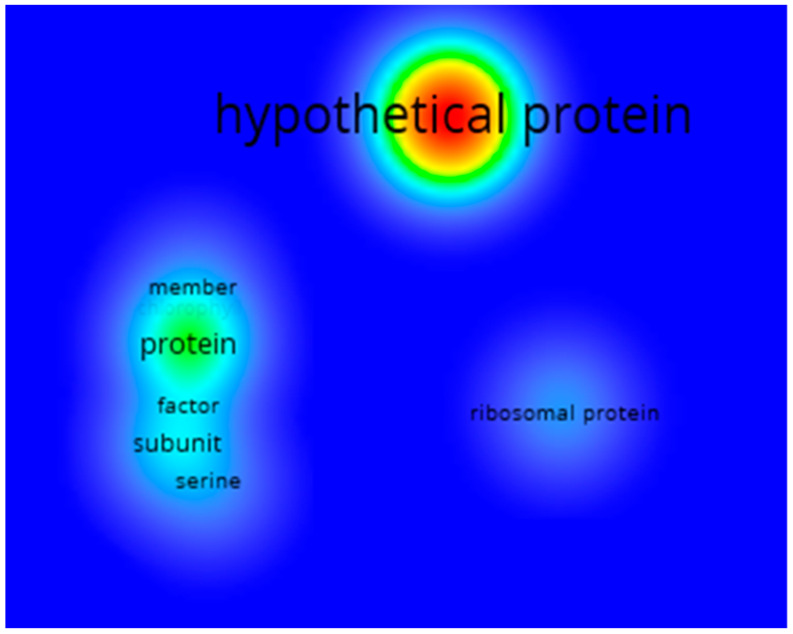
Heat map showing the terms with the highest occurrence describing the 16,807 Chlorophyceae genes related to Se. The red tones represent the most recurring terms, followed by yellow and green, while the less recurring ones are denoted by cyan and finally blue. The font size is also proportional to how often the term can be found.

These results let us know the general behavior of all the genes found in the systematic review. However, to know the behavior patterns of the genes found for Chlorophyceae, it was necessary to analyze the genes found for this class directly. Therefore, Figure 9 shows the keyword co-occurrence analysis of the annotations with the description of the genes for Chlorophyceae. This time the term “hypothetical protein” had the highest occurrence, followed by “transcription factor”, “subunit” and “ribosomal protein”. Based on these results, a large proportion of the genes found for Chlorophyceae, as they belong to hypothetical proteins, are proteins whose existence has been predicted based on sequenced genomes, but which lack insufficient experimental evidence in vivo. Likewise, the other terms allow us to indicate that many genes also involve ribosomal proteins present in the translation process.

On the other hand, it was found that *Dunaliella salina*, a green, halophilic, unicellular alga belonging to the Chlorophyceae class, when exposed to Cadmium (Cd) stress, caused the genes that code for ribosomal proteins to increase significantly. Likewise, exposure to this stress triggered a response that correlated the ribosomes, photosystem, and reactive oxygen species (ROS) elimination pathways. The study with *D*. *salina* is the first to show that ribosomal genes could be the direct target of Cd stress [41]. Similarly, this systematic review shows that genes involving ribosomal proteins could play a key role when Chlorophyceae species are subjected to Se stress. However, this hypothesis can be tested experimentally in future research since interesting variations between strains of this class are likely to occur.

**Figure 10:**
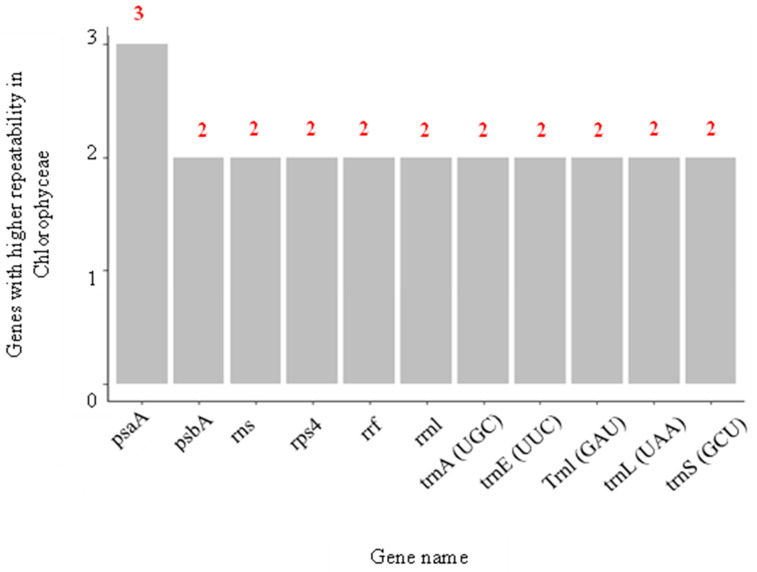
Most frequently repeated genes specifically for Chloropyceae.

Continuing with the behavior of the genes for Chlorophyceae, it was found that, although this class was the one that showed the most significant number of genes associated with Se, their repeatability among the species of that class was minimal (Figure 10). Of the 16,807 genes, the one that showed the highest repeatability was psaA, found three times and involved in the primary electron donation system of photosystem I (PSI). This low repeatability may indicate that the species that make up Chlorophyceae could have a highly variable capacity to adapt to environments with Se. In addition, this could be the reason why when measuring the toxic effect of Se in different microalgae using the effective concentration indicator for 50% growth inhibition (EC_50_), variations in the values are obtained. For example, when comparing the EC_50_ values for different types of microalgae such as *C*. *vulgaris*, *C*. *reinhardtii*, and *H*. *pluvialis*, they are found to be around 73.2, 6.3, and 24.02 mg Se/L in the form of sodium selenite, respectively. Although *C*. *vulgaris*, *C*. *reinhardtii*, and *H*. *pluvialis* are three green algae of the Chlorophyceae class, the capacity of *C*. *vulgaris* to tolerate the toxic effect of high concentrations of Se is almost 11.6 times greater than that of *C*. *reinhardtii* and 3 times higher compared to *H*. *pluvialis* [6].

This finding suggests a high genetic diversity among Chlorophyceae species that could translate into ample opportunities for genetic modification and the creation of new biotechnological products with microalgal biomass involving Se. However, the limited information regarding genome sequencing of the different Chlorophyceae species makes it difficult to identify the primary target genes since they are limited to a few model organisms. However, with the application of advanced omics technologies in this field, it will be possible to explore the Se metabolization capacities and evaluate the quality of producing bioproducts to make them industrially viable [42].

### 3.3. Selenium Accumulation and Metabolism

The metabolic pathways of Se in microalgae are based on two assumptions: (a) Se is further incorporated into amino acids and proteins of microalgae via the metabolic pathway of sulfur (S) assimilation and (b) Se metabolism in higher plants is analogous to microalgae [9]. It should be noted that these microorganisms absorb Se through the soluble forms dominant in aquatic bodies known as selenite or selenate. Additionally, selenate compounds have been reported to be typically more soluble and bioavailable than selenite compounds for marine and freshwater microalgae. While selenite is usually absorbed and accumulated more rapidly compared to selenate [43]. After absorption, selenate and selenite are gradually reduced by the reductive assimilation pathway of S to selenide (Se^2−^), After absorption, selenate and selenite are gradually reduced via the reductive S uptake pathway to selenide, which can be more specifically incorporated into proteins via the SeCys insertion machinery, or gradually metabolized to SeCys and SeMet. SeMet is further volatilized to dimethylselenide (DMSe) or accumulates intracellularly in some microalgae. Selenate has been reported to be metabolized to volatile DMSe in *Chlorella* and SeCys can be further methylated to Se-methylselenocysteine (SeMeSeCys) for accumulation [9]. In addition, metabolization of radiolabeled selenite in the marine microalga *E*. *huxleyi* was reported; after 16 h of culture the microalga incorporated 17% of ^75^Se in the protein fraction and up to 70% as low molecular weight Se compounds, which contained SeMeSeCys but not SeMet or SeCys, suggesting that these seleno-amino acids were rapidly metabolized in *E*. *huxleyi* [44].

On the other hand, selenate, due to its chemical similarity to sulfate, is transported across the plasma membrane by high affinity sulfate transporters. Therefore, it is claimed that selenate and sulfate metabolism is strongly correlated, and Se uptake is negatively correlated with sulfate content in the culture medium. When sulfate is not present in the culture medium, selenate uptake is promoted due to increased transporter activity and reduced competition between selenate and sulfate. On the other hand, selenite is transported by a rapidly saturating specific transport system at low concentrations and a nonspecific transport system at high concentrations, probably involving nitrate and sulfate transporters, which at high concentrations inhibit selenite uptake [9,45,46].

More than 50 selenoprotein families are known in eukaryotes, bacteria, and archaea; in green microalgae, 12 selenoproteins have been identified in *C*. *reinhardtii*. Additionally, in aquatic eukaryotes, selenoproteomes of 26 and 29 genes have been discovered in the marine green microalgae *Ostreococcus lucimarinus* and *Ostreococcus tauri*, respectively, and 16 selenoproteins in the diatom *Thalassiosira pseudonana* [47]. The mechanism of selenoprotein synthesis in microalgae is complex, but similar to that described in humans and mammals. The mechanism involves the translational insertion of SeCys into the catalytic site of selenoproteins using a specific t-RNA (SeCys-tRNA[Ser]Sec), whose anticodon recognizes an opal termination codon (UGA) as a SeCys codon. Recoding of the UGA stop codon is dictated by a SeCys insertion sequence (SECIS) located in the 3′ untranslated region (UTR) of the selenoprotein genes. Genes containing SECIS sequences have sequence similarity between animals and algae and possibly share a common origin [48].

## 4. Conclusions and Future Outlook

Knowledge of Se metabolization and selenoprotein synthesis in algae is only partially understood and needs further investigation. However, it is highlighted that microalgae, due to their reductive metabolism, can physiologically adapt to high Se concentrations by accumulating high amounts of intracellular Se in the form of SeMet and other Se-amino acids. In addition, the accumulation of Se in microalgae generates an alternative that can potentially be used in human and animal nutrition as a source of highly bioavailable Se.

On the other hand, data mining is characterized as a tool that offers the possibility of extracting patterns from large databases, with the purpose of providing evidence of the impact of research results. Consequently, here we found 54,541 genes related to Se metabolization distributed in 160 different classes of organisms. Chlorophyceae (which includes green algae) was the class with the most significant number of genes, with 31.2% of all genes found, followed by Others (16%), Mammalia (13.3%), Teleostei (7.8%), Clostridia (6.9%), Insecta (5.3%), Negativicutes (4.9%), and Aves (4.7%), Gammaproteobacteria (3.1%), Reptilia (1.7%) and Dicotyledons (1.3%). Overall, our findings suggest that a significant portion of the most repeated genes across various species may play a role in translation processes and could potentially encode for synthesized proteins found in the endoplasmic reticulum (ER). These results provide valuable insights into the potential functions of highly repeated genes and their importance in cellular processes, particularly in protein synthesis and folding. Additionally, unlike the other classes, Chlorophyceae presented low gene repeatability across the different species, indicating that these species could have a highly variable capacity for environmental adaptation to Se. This shows a great potential to carry out genetic modifications and create new biotech-based products of Se. However, there is an extensive research gap in molecular biology, which is necessary to expand the genome sequencing of more microalgae species. Thus, we will exploit the microalgae capabilities in Se bioaccumulation for human and animal well-being.

## Data Availability

The datasets used during the current study are available from the corresponding author on reasonable request.
